# High risk of drug toxicity in social isolation stress due to liver dysfunction: Role of oxidative stress and inflammation

**DOI:** 10.1002/brb3.2317

**Published:** 2021-08-01

**Authors:** Maziar Zahir, Siavash Shariatzadeh, Ayda Khosravi, Fatima Ahmed Alshaikh, Parichehr Moradi, Milad Ghaderi, Parsa Farsinejad, Parisa Afraz Louyeh, Saba Ilkhani, Pooria Nakhaei, Amin Taheri, Avid Farhang Fagheh, Reza Akhavan‐Sigari

**Affiliations:** ^1^ Tehran University of Medical Sciences Tehran Iran; ^2^ Department of Pharmacology Shahid Beheshti University of Medical Sciences Tehran Iran; ^3^ Biomedical Engineering Department University of Isfahan Isfahan Iran; ^4^ Department of Neurosurgery University Medical Center Tuebingen Tuebingen Germany

**Keywords:** inflammation, liver, oxidative stress, social isolation stress

## Abstract

**Background**: Previous studies have shown that social isolation stress (SIS) could associate with several systemic diseases; however, the role of SIS on liver dysfunction has yet to be established. This study aimed to investigate the effect of SIS on liver function and possible drug toxicity through liver inflammation and oxidative stress.

**Methods**: Male Naval Medical Research Institute mice in two groups of SIS and control were treated with typical anti‐depressant and anxiolytic agents including fluoxetine, norfluoxetine, desipramine, and imipramine in both groups. Then blood concentrations (or their active metabolites) of these drugs were assessed. Liver function test, including aspartate transaminase (AST), alanine aminotransferase (ALT), total bilirubin, and conjugated bilirubin), oxidative activity, inflammatory cytokines, and the gene expression of cytochrome P450 enzymes were assessed.

**Results**: We observed that the liver enzymes including AST and ALT was slightly higher in SIS animals. The blood concentrations of fluoxetine, norfluoxetine, desipramine, and imipramine were significantly higher in SIS animals. The gene expression of CYP1A2, CYP2A6, CYP2C9, CYP2C29, and CYP2D were significantly decreased in SIS animals. Our results showed that SIS animals had significantly higher level of tumor necrosis factor‐α, interleukin‐1β, and interleukin‐6. SIS could significantly decrease the activity of antioxidant agent (Glutathione).

**Conclusion**: We hypothesized that SIS could induce liver dysfunction and decrease the rate of drug clearance through liver inflammation and oxidative stress; therefore, the blood concentration of anti‐depressant/anxiolytic agents should closely monitor in SIS due to the high toxicity of these agents.

## Graphical abstract

In the current study, we hypothesized that social isolation stress could induce liver dysfunction and decrease the rate of drug clearance through liver inflammation and oxidative stress; therefore, which could lead to drug toxicity with anti‐depressants and anxiolytics.

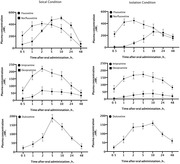

## 1 INTRODUCTION

Chronic exposure to social and psychological stress has been postulated as a detrimental factor for health and consequently associated to lifestyle‐related diseases such as hyperglycemia, hyperinsulinemia, obesity, cardiovascular disease, and cancer(Ader & Cohen, 1993, McEwen & Stellar, 1993, Raikkonen et al., 1996, Chrousos & Gold, 1998, Chandola et al., 2006, Brunner et al., 2007, Danese et al., 2009). Accordingly, it has been shown that social isolation stress (SIS), as a valid paradigm of depression, anxiety, and memory impairment, is strongly associated with increased prevalence of cardiovascular diseases, colon cancer, and liver cancer. (Grant et al., 2009, Wu et al., 2000, Wu et al., 2000). Although the devastating effects of SIS on many organ systems have been thoroughly investigated, the effect of SIS on liver dysfunction and the possible underlying mechanism is not investigated yet.

For many years, it was believed that stress is responsible for diminished hepatic perfusion through activation of vasospasm mechanisms and it was postulated that the subsequent centrilobular hypoxia would lead to liver damage (Hirose et al., 1961, Kaplan & Wheeler, 1983). With further understanding of stress mediators in the last two decades, studies investigating the role of stress on the emergence and expansion of liver injury during acute and chronic hepatic diseases have reached a new perspective (Swain, 2000, Chida et al., 2006). Psychosocial stress is assumed to deteriorate prior hepatic inflammation by over‐activation Hypothalamic‐pituitary‐adrenal (HPA) axis and directly inducing the release of pro‐inflammatory cytokines such as interleukin‐6 (IL‐6) and tumor necrosis factor‐α (TNF‐α) (Swain, 2000, Tjandra et al., 2000). In addition, escalated oxidative markers and compromised hepatic antioxidant defense system has been observed as an inevitable outcome of established chronic stress (Madrigal et al., 2001, Kaushik & Kaur, 2003, Djordjevic et al., 2011). Despite the ample research done to discover the pathophysiology of liver dysfunction in SIS, the probability of drug toxicities as an outcome of hepatic inflammation and dysfunction has not been evaluated.

Drug toxicity can have catastrophic consequences, such as critical organ failures (Pereira et al., 2011, Huh et al., 2012), and death; in the case of antidepressant drugs, this dilemma is signified due to their narrow therapeutic window and severe cardiotoxic and neurotoxic side effects in overdose (Grundemar et al., 1997). The key enzyme in their metabolism is cytochrome P450 (CYP450) (Xu et al., 2016, Westphal & Brogard, 1997). According to the above‐mentioned evidence, any decline in liver function can cause increased serum levels of these drugs and subsequent to overdose or toxicity. Furthermore, it has been well‐known that some of these drugs might induce hepatotoxicity directly, such as the selective serotonin reuptake inhibitor, fluoxetine (Capella et al., 1999, Friedenberg & Rothstein, 1996). This combined mechanism of direct toxicity and lowered excretion can dramatically increase the risk of overdose and toxicity.

Djordjevici et al. showed that chronic stress might result in high oxidative stress level in liver tissue, and it could be hypothesized that imbalance in oxidative stress might result in liver inflammation and dysfunction (Djordjevic et al., 2010). The main purpose of this study was to evaluate the effects of SIS on hepatic inflammation and dysfunction and possible consequence of drug toxicity due to liver failure in animal model. We quantified the serum levels of various antidepressant drugs to estimate the rate of their activation and elimination. In this regard, we used fluoxetine hydrochloride, imipramine hydrochloride, and duloxetine hydrochloride to evaluate the drug toxicity in SIS, due to high metabolization rate by different CYP450 subtypes and also close bioavailability (Wyska, 2019); also, based on previously published reports, these medications are the most prescribed drugs in United State in the clinical settings of depression and anxiety (Chen et al., 2008). In order to evaluate the extent of hepatic inflammation, we measured the serum levels of alanine transaminase (ALT), aspartate transaminase (AST), alkaline phosphatase (ALP), and bilirubin. We also measured serum levels of interleukin‐1 (IL‐1), interleukin‐6 (IL‐6), TNF‐α, and the gene expression of CYP450 in order to detect the extent of inflammation and the hepatic enzymatic capacity, respectively.

## 2 MATERIAL AND METHODS

### 2.1 Animals and housing conditions

Total of 30 male Naval Medical Research Institute mice aged 21–25 days and weighing 10–12 g were used in this study. Animals were housed in two different conditions including social condition (SC, *n* = 10) and isolated condition (IC, *n* = 20). All animals were kept under standard laboratory conditions i.e. temperature: 22 ± 2°C, humidity: 50 ± 10%, 12‐h light‐dark cycle, and ad‐libitum access to food and water for a period of 5 weeks. Socially conditioned mice were placed in Plexiglas boxes (25 cm × 25 cm × 15 cm) (6 mice per cage) and IC animals were placed individually in Plexiglas boxes (24 cm × 17 cm × 12 cm) (Haj‐Mirzaian et al., 2017, Haj‐Mirzaian et al., 2020). In order to diminish handling and social interaction cages of IC animals were cleaned weekly by the same experimenter. All behavioral tasks were carried out between 10:00 a.m. and 2:00 p.m.

### 2.2 Drugs and chemicals

Fluoxetine hydrochloride, Imipramine hydrochloride, and Duloxetine hydrochloride were obtained from Sigma‐Aldrich (US). Tris‐hydroxymethyl aminomethane, 2‐thiobarbituric acid, 5, 5‐dithio nitrobenzoic acid (DTNB), and trichloroacetic acid (TCA) were purchased from Sigma‐Aldrich (US). Pyridoxine hydrochloride, 2‐pyrrolidone‐5‐carboxylic acid, *N*‐acetyl cysteine, 1, 1‐3, 3‐tetramethoxypropane, and glutathione (GSH) powder were purchased from Sigma‐Aldrich (USA). The kits for liver biochemistry assay (ALT, AST, alkaline phosphatase (ALP), and total bilirubin) were obtained from Sigma‐Aldrich (US).

### 2.3 Sample acquisition and preparation

Blood was drawn by tail vein and added to sterile tubes allowed to clot then centrifuged at 3000 rpm for 10 min to obtain serum. Serum AST, ALT, ALP levels, and total bilirubin were measured and the results were expressed in U/L (total bilirubin was expressed as g/L). After collecting blood samples, the liver of mice carefully separated and washed with ice‐cooled saline. All sections of each mouse liver were frozen immediately in liquid nitrogen and then stored at −80°C.

### 2.4 Experimental design

Both socially isolated and normal mice were randomly divided into four groups of at least six animals. The treatment groups were as follow: the first group received oral normal saline (5 ml/kg) through a gastric tube. The second group received a single oral fluoxetine (15 mg/kg diluted in water), the third group received imipramine (20 mg/kg diluted in water), and fourth groups received a single oral dose of duloxetine (10 mg/kg diluted in water). All doses were chosen based on previous studies; and it has been shown that all of these antidepressants were effective in SIS (fluoxetine (Friedenberg & Rothstein, 1996), imipramine (Ramirez et al., 2015), and duloxetine (Xu et al., 2016)). Blood samples were collected 0.5, 1, 2, 5, 10, 24, 36, and 48 h after drug administration in each animal (the time interval was chosen based on previously published manuscript (Sager et al., 2014, Baœ et al., 2004)). In addition, collected blood samples in saline‐treated animals were considered as normal group and was sent for biochemistry analysis of ALT, AST, and ALP. Also, saline‐treated animals were sacrificed at the end of the experimental tasks and liver tissue resected and stored in −80°C for further analysis.

### 2.5 Pharmacokinetic study

Heparinized blood samples were collected by means of a butterfly catheter before and at the following times post‐administration: 0.5, 1, 2, 5, 10, 24, 36, and 48 h in both socially isolation and normal animals (the time intervals were chosen based on previous studies and also the drugs’ characteristics) (Sager et al., 2014, Baœ et al., 2004). After centrifugation (1500 g, 10 min, 4°C), the supernatant was separated and used for further analysis. The separated plasma was stored at −20°C until the assay. Standard breakfast, lunch, and dinner were allowed, respectively, 2, 5, and 8 h after drug intake.

Fluoxetine and norfluoxetine, duloxetine, imipramine, and desipramine plasma levels were determined using a validated high‐performance liquid chromatography method after a solid phase extraction procedure. The limit of quantification was equal to 0.625 ng/ml and the linearity was achieved between 0.625 and 20 ng/ml for fluoxetine and norfluoxetine.

### 2.6 Assay of oxidative stress and inflammation

The liver GSH contents were measured by determining nonprotein sulfhydryl contents with the Ellman's reagent. Two hundred milligram of the liver was weighed and homogenized in 8 ml of cooled ethylenediaminetetraacetic acid solution (0.02 M) in an ice bath. Then, 5 ml of liver homogenate was transferred to new tube and added 4 ml of distilled water and 1 ml of 50% TCA. The mixture was shaken vigorously for 10 min and then centrifuged (15 min at 4°C). Then, 2 ml of supernatant was added to 4 ml of Tris buffer (pH 8.9) and 100 μL of DTNB solution (0.01 M in methanol). The samples were shaken to obtain a homogeneous mixture. The solution absorbance was read within 5 min of the addition of DTNB at 412 nm against a reagent blank with no homogenate (Haj‐Mirzaian et al., 2019). In addition, TNF‐α, IL‐6, and interlukin‐1β (IL‐1β) were spectrophotometrically analyzed according to the instructions of ELISA kit (Biosciences, USA).

### 2.7 Real‐time PCR analysis

Liver was resected and immediately frozen in liquid nitrogen, then stored at −80°C. In the first step total RNA was extracted from thoracic aorta using Trizol reagent (Invitrogen, Cergy Pontoise, France). Alterations in the mRNA levels of genes were determined using qRT‐PCR after the reverse transcription of 1 μg of RNA from each sample using PrimeScript RT reagent kit (Takara Bio Inc., Otsu, Japan). qRT‐PCR was completed on a light cycler device (Roche Diagnostics, Mannheim, Germany) using SYBR Premix Ex Taq technology (Takara Bio). Sequences of primers were designed based on previous reports that are shown in Table 1. Thermal cycling conditions included an initial activation step for 30 s at 95°C afterward 45 cycles as well as a denaturation step for 5 s at 95°C and a combined annealing/extension step for 20 s at 60°C (Haj‐Mirzaian et al., 2019). Melting curve analysis was performed to certify whether all primers yielded a single PCR product.

**TABLE 1:** Sequences of primers used for quantitative RT‐PCR on mice

**Gene name**	**Primers (forward and reverse 5’ to 3’)**
**CYP1A2**	F: 5’‐AGTACATCTCCTTAGCCCCAG‐3’
	R: 5’‐GGTCCGGGTGGATTCTTCAG‐3’
**CYP2A6**	F: 5’‐GTGGTCCTGGAGGCATTCAA‐3’
	R: 5’‐CAGTACTGGGTAAGACCACTGA‐3’
**CYP2C9**	F: 5’‐TTCTAGGTGTGTTTCTGGGGC‐3’
	R: 5’‐AACACCACAGCAGGATTCCTCA‐3’
**CYP2C29**	F: 5’‐ATCTGGTCGTGTTCCTAGCG‐3’
	R: 5’‐AGTAGGCTTTGAGCCCAAATAC‐3’
**CYP2D**	F: 5’‐CCCATCTTTGAGCATCTTGGT‐3’
	R: 5’‐ GCCCAGCCTGAGTAGTGAAG‐3’

### 2.8 Statistical analysis

One‐way ANOVA, two‐way repeated measure ANOVA and T‐test analyses were used to analyze the data in the current study (GraphPad Prism version 7); and all data were normally distributed. *p*‐value < 0.05 was the critical criterion for statistical significance. We used Bonferroni P value adjustment for RT‐PCR result and *p*‐value less than 0.01 was consider as significance level in RT‐PCR results. In addition, the sample size was determined using version 3 of G*Power software, considering the study's power of 0.8 and *α* = 0.05.

### 2.9 Ethics

Our study was in accordance with the National Institute of Health (NIH) Guidelines for the Care and Use of Laboratory Animals (HHS publication 85‐23, 1985), legislation for the protection of animals used for scientific purposes (Directive 2010/63/EU).

## 3 RESULTS

In the first part of results section, we showed that SIS could significantly impact on liver enzymes including AST, ALT, and ALP. Table 2 shows that the plasma concentration of AST (*p* < .001, *t* = 6.18, df = 8) and ALT (*p* < .001, *t* = 8.09, df = 8), as well as ALP (*p* < .001, *t* = 25.5, df = 8) significantly increased in socially isolated animals in comparison to normal animals. Also, t‐test analysis failed to show any significant difference between socially isolated and normal condition animals in regard of total bilirubin and conjugated bilirubin (*p* > .05, Table 2). In addition, we assessed the liver inflammation. Our results showed that SIS significantly increased the level of TNF‐α (*p* < .001, *t* = 9.89, df = 8), IL‐6 (*p* < .001, *t* = 18.70, df = 8), and IL‐1β (*p* < .001, *t* = 18.47, df = 8) in comparison to SC animals (Table 3). Also, we observed that SIS significantly decreased the activities of oxidative stress marker enzymes (GSH) in comparison to normal mice (*p* < .001, *t* = 27.19, df = 8).

**TABLE 2:** Evaluation of liver enzymes in normal and socially isolated animals

**Items**	**Social condition**	**Isolation condition**	***p*‐value**
AST (U/l)	48.8 ± 1.9	81.3 ± 11.6	<0.001
ALT (U/l)	37.1 ± 2.1	70.2 ± 8.9	<0.001
ALP	73.1 ± 6.9	188.5 ± 7.4	<0.001
Total bilirubin	1.67 ± 0.32	1.79 ± 0.53	*p* = .773
Conjugated bilirubin	0.39 ± 0.12	0.48 ± 0.19	*p* = .391

**TABLE 3:** Evaluation of the inflammatory cytokines and oxidative stress activity in liver samples of normal and socially isolated animals

**Items**	**Social condition**	**Isolation condition**	***p*‐value**
TNF‐α (pg/ml)	206.1 ± 33.1	541.5 ± 68.2	<0.001
IL‐6 (pg/ml)	79.8 ± 11.1	266.7 ± 19.4	<0.001
IL‐1β (pg/ml)	188.2 ± 21.7	478.9 ± 27.7	<0.001
GSH (ng/L)	698.2 ± 22.0	337.5 ± 19.9	<0.001

**FIGURE 1:**
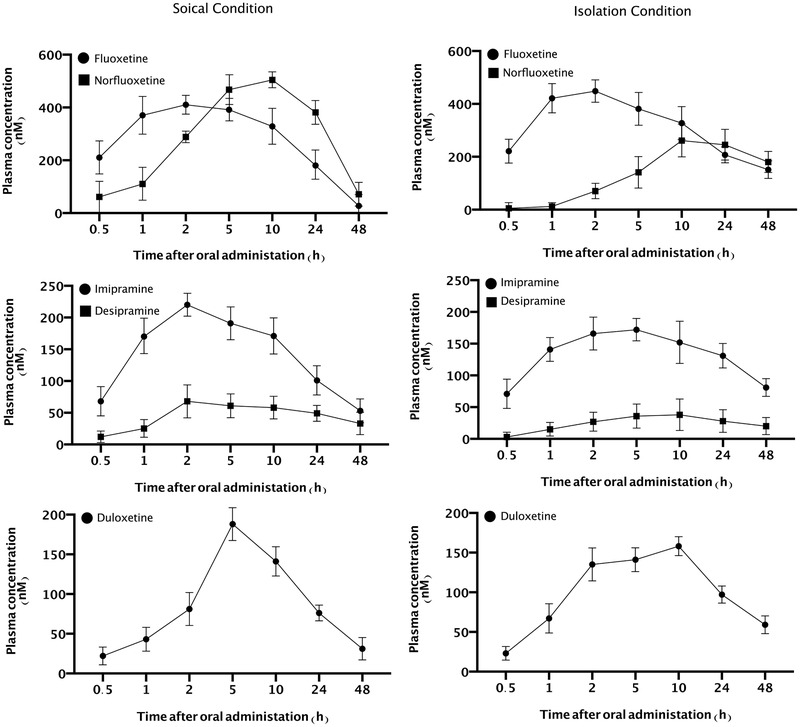
Evaluation of plasma concentration of fluoxetine, imipramine, duloxetine and their metabolites (norfluoxetine and desipramine) 0.5, 1, 2, 5, 10, 24, 48 h after oral administrations in both IC and SC animals. Values are expressed as the mean ± S.E.M (*n* = 6) results were analyzed using two‐way ANOVA repeated measure followed by Tukey's post hoc test

In the next part of the results section, we evaluated the plasma concentration of fluoxetine, norfluoxetine, imipramine, desipramine, and duloxetine in both normal and socially isolated animals in seven intervals (0.5, 1, 2, 5, 10, 24, and 48 h after single dose oral administrations). Two‐way ANOVA analysis showed that SIS could significantly impact on the plasma concentration of fluoxetine (F (6, 30) = 225.3, *p* < .001, Figure 1) and norfluoxetine (F (6, 30) = 222.7, *p* < .001, Figure 1) in comparison to normal animals. Our results showed that the plasma level of fluoxetine reached the highest level 2 h after oral administration and norfluoxetine reached the highest level 10 h after oral administration in normal animals. In addition, the plasma levels of both norfluoxetine and fluoxetine significantly decreased after 48 h in comparison with the plasma levels at 2 h after administration in normal mice (*p* < .001). Furthermore, no significant difference was observed between the level of both fluoxetine and norfluoxetine at 48 and 0.5 h after oral administration in SC group (*p* > .05). On the other hand, results obtained from IC groups showed that the maximum plasma levels of fluoxetine and norfluoxetine were observed at 2 and 10 h after oral administration, respectively. We observed higher levels of fluoxetine and norfluoxetine at 48 h after administration in comparison to 0.5 h (*p* < .01).

Two‐way ANOVA analysis showed that SIS could significantly impact on the plasma concentration of imipramine (F (6, 30) = 169.9, *p *< .001, Figure 1) and desipramine (F (6, 30) = 103.1, *p *< .001, Figure 1) in comparison to normal animals. Our results showed that the plasma level of imipramine reached the highest level 2 h after oral administration and desipramine reached the highest level 10 h after oral administration in normal animals. Besides, the plasma level of both imipramine and desipramine significantly decreased after 48 h in comparison to the plasma level at 2 h after administration in normal mice (*p *< .001). In addition, no significant difference was observed between the level of both imipramine and desipramine at 48 and 0.5 h after oral administration in SC group (*p *> .05). Results obtained from socially isolated animals demonstrated that the maximum plasma levels of imipramine and desipramine were observed at 2 and 10 h after oral administration, respectively. We observed higher levels of imipramine and desipramine at 48 h after administration in comparison to 0.5 h in IC group (*p *< .05).

Evaluation of plasma concentration of duloxetine showed that SIS could significantly impact on the plasma concentration of duloxetine (F (6, 30) = 343.3, *p *< .001, Figure 1) in comparison to normal animals. Our results demonstrated that the plasma level of duloxetine reached the highest level 5 h after oral administration. In addition, the plasma level of duloxetine in socially conditioned mice significantly decreased after 48 h in comparison to the plasma level at 5 h after administration (*p *< .001). Furthermore, no significant difference was observed between the level of duloxetine at 48 and 0.5 h after oral administration in SC group (*p *> .05). Results obtained from socially isolated animals demonstrated that the maximum plasma level of duloxetine was observed at 10 h after oral administration. We observed higher levels of duloxetine at 48 h after administration in comparison to 0.5 h in IC group (*p* < .001).

**FIGURE 2:**
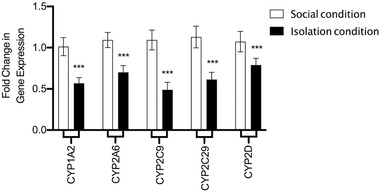
Evaluation of gene expression of CYP1A2, CYP2A6, CYP2C9, CYP2C29, and CYP2D in both SC and IC animals. Values are expressed as the mean ± S.E.M (*n* = 6) results were analyzed using one‐way ANOVA followed by Tukey's post hoc test

In the final step of the results section, one‐way ANOVA analysis showed that SIS could significantly impact on gene expression of cytochrome P450 (CYP) 1A2, 2A6, 2C9, 2C29, and 2D (F (9, 39) = 30.11, *p *< .001, Figure 2) in liver tissue samples. Tukey's analysis showed that the gene expression of CYP1A2, CYP2A6, CYP2C9, CYP2C29, and CYP2D significantly decreased in IC animals in comparison to normal animals (*p* < .001).

## 4 DISCUSSION

In the current study, we observed that the liver enzymes including AST and ALT was slightly higher in SIS animals. The blood concentrations of fluoxetine, norfluoxetine, desipramine, and imipramine were significantly higher in SIS animals. The gene expression of CYP1A2, CYP2A6, CYP2C9, CYP2C29, and CYP2D were significantly decreased in SIS animals. Our results showed that SIS animals had significantly higher level of TNF‐a, IL‐1β, and IL‐6. SIS could significantly decrease the activity of antioxidant agent (Glutathione, GSH).

Social isolation stress paradigm has been proved as a valid animal model of psychosocial stress; capable of inducing psychiatric and metabolic disorders such as irregular pattern of weight gain, increased visceral fat accumulation, elevated incidence of coronary heart disease, and disrupted HPA axis activity (Nonogaki et al., 2007, Ros‐Simó & Valverde, 2012, Sakakibara et al., 2012, Sun et al., 2014, Das & O'Keefe, 2006). Moreover, social isolation has been established as a major risk factor of liver pathologies.

The exact mechanism of how social stress can affect liver pathologies is still obscure. Formerly, activation of the locus coeruleus‐norepinephrine system and the resultant catecholamine release were assumed to induce vasospasm and the consequent centrilobular ischemia seemed responsible for hepatic inflammation and injury (Hirose et al., 1961, Kaplan & Wheeler, 1983, Swain, 2000). Recently, stress mediators have gained attention as a pivotal effector in liver pathologies (Chida et al., 2006). The HPA axis, systemic sympathetic and adrenal medullary systems are the main constituents of the stress system. They maintain homeostasis in both basal and stressful situations (Chrousos & Gold, 1992, Chrousos, 1992). They are activated through various mechanisms. Several inflammatory cytokines such as, TNF‐α, IL‐1, and IL‐6 are known to profoundly influence and activate this stress system (Chrousos & Gold, 1992, Chrousos, 1992, Sawchenko et al., 1993). According to our data higher levels of TNF‐α, IL‐1, and IL‐6 was observed in IC mice; indicating an overwhelming inflammatory response and immense activation of stress system in this group.

In order to evaluate the extent of liver dysfunction, we measured serum transaminases, enzymes which rise in the course of hepatic inflammation and toxic liver injuries and can be used as an accurate indicator of disintegration in cytoplasmic and/or mitochondrial membranes (Feldman et al., 2006). Increased ALT level is a sensitive indicator of hepatic disease and is more specific for liver injuries compared to AST levels because of its cytoplasmic location. Liver cells store higher levels of AST than ALT but ALT is mainly contained in the cytoplasm where its concentration exceeds that of AST, whilst AST is present in both cytoplasmic and mitochondrial locations (Feldman et al., 2006). Previous studies have shown that superimposed depression in patients with chronic viral hepatitis B is significantly correlated with increased serum hepatic transaminases (Kunkel et al., 2000). Moreover, a positive correlation was seen between psychosocial stress and serum (ALT) values in cirrhotic patients (Nagano et al., 2004).

The results of the current study indicated that mice exposed to social isolation stress showed liver dysfunction. Our results revealed a significant rise in the serum levels of ALT, AST, and ALP in socially isolated mice; suggesting a simultaneous hepatocellular and cholestatic pattern of injury in this group. Moreover, a slight rise in total serum bilirubin was also detected, further supporting a cholestatic disease. Our results regarding hepatocellular injury were aligned with those of a previous study which reported significantly increased ALT and AST activity in fluoxetine treated animals (Inkielewicz‐Stępniak, 2011). Additionally, our data unveiled a simultaneous cholestatic injury and this finding was first of its kind in clinical studies. It has to be noted that the increased level of AST in animals treated with fluoxetine can be a result of fluoxetine‐induced loss of mitochondrial membrane function (Feldman et al., 2006).

Another important intracellular mechanism of liver injury is the depletion of the cells from glutathione (GSH), a major anti‐oxidant molecule capable of detoxifying hydrogen peroxide and lipid peroxides (Gupta et al., 2005). Our results demonstrated that the level of glutathione was meaningfully decreased in socially isolated mice, suggesting a lowered capacity of the anti‐oxidant defense system in this group. In addition, its noticeable that 4‐trifluoromethylphenol, one of fluoxetine's metabolites, has been shown to decrease GSH levels directly (Thompson et al., 2000).

It has been long known that antidepressants go through extensive biotransformation in the liver. The main enzyme in charge of their metabolism is cytochrome P450 (Caccia, 1998). Interestingly, cytochromes function is known to be severely disrupted by antidepressant drugs and their active metabolites (Caccia, 1998). We assessed the serum levels of antidepressants and the gene expression of cytochrome P450 to evaluate the possibility of drug toxicity due to hepatic dysfunction in the course of SIS, Five major antidepressant drugs (fluoxetine, norfluoxetine, duloxetine, imipramine and desipramine) were carefully chosen based on their close bioavailability and a wide range of metabolization enzyme and also high prescription rate in US. The bioavailability of these drugs is nearly the same (all ranging from 50% to 70%) which makes them suitable for comparison (van Harten, 1993, Abernethyl et al., 1984). Additionally, these drugs have been shown to inhibit various cytochrome subtypes. For instance, fluoxetine and duloxetine inhibit CYP2D, norfluoxetine is a moderate inhibitor of CYP1A2 (Hemeryck & Belpaire, 2002, Skinner et al., 2003), and imipramine and desipramine are known to exert inhibitory effects on CYP2D6, CYP2C9, and CYP2C29 in mice (Shin et al., 2002). In this regard, we measured the gene expression of cytochrome P450 (CYP) 1A2, 2A6, 2C9, 2C29, and 2D. Their expression was significantly decreased in IC mice; emphasizing the diminished enzymatic capacity in this group. The combined effect of lowered gene expression and enzymatic inhibition caused by these drugs and their end‐metabolites, emphasizes the vulnerability of hepatic metabolic system and the necessity of lowering administration dose to avoid any probable drug toxicity.

Another piece of meaningful evidence was the altered serum levels of anti‐depressant drugs in socially isolated mice. The plasma level of fluoxetine and norfluoxetine were raised in a much slower rate in IC mice than SC mice. Additionally, the peak serum level of norfluoxetine was significantly lower in socially isolated mice, suggesting a decreased metabolic function of the liver. In addition, the elimination rate of fluoxetine and norfluoxetine was meaningfully lower in socially isolated mice. These findings are indicative of an impaired hepatic function and declined hepatic enzymatic capacity. Our findings are parallel to those of Schenker et al. who concluded that the elimination and plasma clearance of fluoxetine and norfluoxetine is severely disrupted in patients with stable alcoholic cirrhosis (Schenker 1 et al., 1988). We also measured the serum levels of imipramine, desipramine and duloxetine in both groups. In IC mice the peak serum level of these drugs was lower than SC mice but their elimination rate was significantly lower resulting in an increased imipramine and duloxetine level after 48 h. On the basis of our findings a less frequent administration or lower dose of antidepressant drugs is recommended in depressive disorder patients in order to avoid drug toxicity and overdose since many of them might have varying degrees of hepatic failure. Finally, in clinical view, most cases with major depression disease, which induced by loneliness, typically treated with several anti‐depressant/anxiolytic plus benzodiazepine. Thus, in this situation patients might be prone to drug toxicity and overdose than a single anti‐depressant/anxiolytic treatment; and the blood concentration of anti‐depressant/anxiolytic drugs or their symptoms should carefully monitor.

The major limitation of the current study was that our research did not provide any proper positive control to SIS. Thus, it could not be concluded that liver dysfunction and drug toxicity is presentenced following SIS or any kind of chronic stress. Therefore, further studies should be established to investigate the liver function following other animal models of depression and anxiety. Also, the treatment regimen consisted of a single dose of antidepressant drugs. According to previous data, a standard treatment duration for depressive disorders is at least 2 weeks (Blier, 2009). Contrastingly, as mentioned above, antidepressants and their active metabolites have been long known to inhibit cytochrome p450 isoforms meaningfully (Hemeryck & Belpaire, 2002, Skinner et al., 2003, Shin et al., 2002, Caccia, 1998). In our study the main purpose was to evaluate the probability of overdose and drug toxicity and we did not intend to evaluate treatment outcomes so we used a single dose treatment in order to exclude the inevitable inhibitory effect of antidepressants on cytochrome p450.

## 5 CONCLUSION

In conclusion, our results indicated that social isolation might induce liver dysfunction. We also demonstrated that inflammatory cytokines increase meaningfully in the course of liver disease. According to our data, the capacity of the anti‐oxidant system was reduced significantly in IC mice. Moreover, according to our data, the gene expression of CYP450 was remarkably decreased in all subtypes, indicating a diminished metabolic capacity of the liver. Our measurements also revealed that it took longer time for antidepressant drugs to reach peak serum levels in IC mice and it took much longer time for the liver to clear them up from the circulation so close monitoring of the serum levels of antidepressant drugs is mandatory in order to avoid any possible toxicity or side effect.
